# Research on the human virome: where are we and what is next

**DOI:** 10.1186/s40168-016-0177-y

**Published:** 2016-06-24

**Authors:** Shimian Zou, Lis Caler, Sandra Colombini-Hatch, Simone Glynn, Pothur Srinivas

**Affiliations:** National Heart, Lung, and Blood Institute (NHLBI), National Institutes of Health (NIH), 6701, Rockledge Drive, Room 9144, Bethesda, MD 20892-7950 USA

**Keywords:** Microbiome, Virome, Heart, Lung, Blood, HIV

## Abstract

The National Heart, Lung, and Blood Institute (NHLBI) of the National Institutes of Health convened a Working Group on the Microbiome in Cardiovascular, Pulmonary and Hematologic Health and Diseases from June 25, 2014, to June 26, 2014. The Working Group’s central goal was to define what major microbiome research areas warranted additional study in the context of heart, lung, and blood (HLB) diseases. The Working Group identified studies of the human virome a key priority.

## Introduction

The Human Microbiome (http://hmpdacc.org/overview/about.php, last accessed January 11, 2016) comprises the bacteria, viruses, and other microorganisms living in association with the human body [1–3]. To define what major microbiome research areas warranted additional study in the context of heart, lung, and blood (HLB) diseases, the National Heart, Lung, and Blood Institute (NHLBI) of the National Institutes of Health convened a Working Group on the Microbiome in Cardiovascular, Pulmonary and Hematologic Health and Diseases from June 25, 2014, to June 26, 2014. The objectives of the Working Group included: (1) evaluation of the current state of knowledge on the role of microbiome in cardiovascular, pulmonary, or hematologic health and disease outcomes, (2) assessment whether this knowledge can be used to identify the means to modulate the microbiota or its dependent processes to prevent disease and promote health, and (3) identification of next steps to help move the research forward. The Working Group identified studies of the human virome a key priority.

The composition of the human virome includes viruses that infect human cells, ancient virus-derived elements inserted in our chromosomes, and bacteriophages that infect a broad array of bacteria that inhabit us. As a recent report highlighted, there is a disparity relative to the need in the level of support for virome research [4]. Despite advances in sequencing technology, there are still very few studies addressing non-bacterial components of the microbiome including the virome [5]. Challenges include lack of common markers for viruses in contrast to bacteria, the heterogeneity of the virome elements, difficulties working with low biomass samples, confounding by host DNA background, inadequate bioinformatic tools for analysis of the virome, and a lack of robust and curated virome databases. This report aims at summarizing the current state of the science and examining the next steps in this exciting field.

## Current status of human virome research

The description of the human virome, when compared to the now well-described bacterial microbiome, is relatively recent and rather limited. Currently, a few virome studies suggest that viruses may have either beneficial or detrimental effects on human health, depending on their interactions with the host, other viruses, and bacteria [6–16]. Studies have shown that salivary, gastrointestinal, and respiratory tract viromes are distinct, indicating that the local environment plays a fundamental role in shaping human viromes [17]. These studies also suggest that these viruses may have a role in helping shape the microbial diversity in the human oral cavity [17]. Gut bacteria play a fundamental role in human health and have been implicated in a number of pathophysiological processes, including obesity, inflammatory bowel disease (IBD), and cardiovascular disease (CVD) [18]. Studies have suggested that regulation of microbial diversity in the gastrointestinal tract and systemic circulation by the virome could have far-reaching implications. For example, it was observed that specific expansion of Caudovirales (bacteriophages) in Crohn’s disease has been associated with decreased bacterial diversity, supporting the idea that the virome may contribute to intestinal inflammation and bacterial dysbiosis [19]. Abeles and Pride have shown that the bacteriophages in the gut could affect the bacterial flora that host them [20]. Hence, it is possible that they could impact bacterial function as well. Tang and Hazen have shown that a subset of gut bacteria are involved in the formation of trimethylamine, a metabolite linked to atherosclerosis, and it is possible that bacteriophages could modulate this process [21].

A very limited number of studies have focused on the virome of the lung and respiratory tract. Many respiratory infectious diseases are caused by bacteria and/or viruses that live in a complex interactive environment [22]. Disturbances of this environment by colonization of new entry species can lead to overgrowth and invasion of pathobionts (defined as “any potentially pathological (disease-causing) organism which, under normal circumstances, lives as a symbiont”), leading to disease. Viral presence alters the host epithelium (first line of host defense) rendering it more susceptible to bacterial infection [23]. Indeed, research has suggested that respiratory viruses and the consequent response of the innate immune system could also drive the development of asthma and chronic obstructive pulmonary disease (COPD) [9]. Further, studies of the DNA virome within the respiratory tract following lung transplantation shows complex anellovirus populations in transplant recipients, with multiple concurrent variants. These high anellovirus loads correlated with dysbiotic bacterial communities in allograft bronchoalveolar lavages. Although it has been known for some time that long-term lung transplant outcomes have been linked to prior infection with several viruses, such as cytomegalovirus and community-acquired respiratory viruses, this research example highlights the importance and need to better understand the human virome, as it may unravel unique factors and mechanisms influencing both viral and bacterial microbiota that ultimately may dictate disease outcomes [24].

Many viruses can persist in cells such as hematopoietic cells and vascular endothelial cells, and such viruses can influence the host in profound ways independent of classical viral disease [1, 25]. The immune system is continuously stimulated by chronic systemic viruses, and this aspect of host-microbiome interactions appears specific to the virome. The virome is considered one of the drivers of idiopathic systemic inflammation that has been linked to many of the most severe public health problems, including cardiovascular diseases [25]. Alternatively, some viruses may be protective; for example, GB virus C or hepatitis G virus (HGV) may have a protective effect against human immunodeficiency virus (HIV)-associated disease [15]. Cytomegalovirus, a highly prevalent virus, appears to promote recipient T cell immunity following reduced-intensity T cell-depleted hematopoietic stem cell (HSC) transplantation [16].

While these initial reports highlight the potential role of the virome in health, resilience, and disease, the basic molecular and physiological mechanisms underlying these effects remain largely unknown. Do viruses have a role in cellular networks, cell-cell interactions, developmental pathways, or interactions with commensal bacteria that can potentiate or lead to disease, and if so what are the molecular mechanisms? Do viral chromosomal insertions contribute to host adaptation? Can specific viruses prime our immune system and how? How do specific vascular endothelial cell viromes affect the interactions between the endothelium and blood elements? Are viruses involved in normal cell homeostasis? Can they, through immune and inflammatory mediators, explain some of the variations in phenotypes among persons sharing known genetic mutations such as cystic fibrosis or sickle cell disease?

Limited studies have also shown the potential importance of the human virome in the context of HIV infection. Other than the protective effect of GB virus C as indicated above [15], pathogenic simian immunodeficiency virus infection appears to be associated with expansion of the enteric virome and acquired immunodeficiency syndrome (AIDS) alters the commensal plasma virome [26–28]. Recent work shows that the virome can chronically stimulate the immune system, potentiating ongoing inflammatory processes [25]. HIV-infected adults have an excess risk of cardiovascular, liver, kidney, bone, and neurologic diseases. Many markers of inflammation are elevated in HIV disease and strongly predictive of morbidity and mortality. Understanding the role of the virome in inflammation could potentially permit the identification of strategies to prevent or decrease the development of comorbidities in HIV patients [29]. Research questions of interest include: How does HIV interact with the rest of the virome and the microbiome? What is the role of genetic determinants in affecting the interactions between HIV and the virome? What are the mechanisms by which interactions between HIV and the virome contribute to heart, lung, and blood co-morbidities as well as HIV pathogenesis and chronic inflammation? What is the role of the virome in heart, lung, and blood health as well as other co-morbidities in the context of treatment of HIV infection with anti-retroviral therapy?

## What is next?

The NIH Human Microbiome Project (HMP) [30] has allowed us to better appreciate the role of bacteria in human health and has also led to the realization of the potential importance of the virome for heart, lung, and blood health and resilience as well as diseases. The advances facilitated through HMP efforts, such as metagenomic sequencing and computational analysis of large quantities of sequence data, can be leveraged for human virome research. However, the unique challenges facing virome research, such as the lack of a universal viral molecular marker (similar to bacterial 16S rRNA) and the heterogeneity of the virome elements, highlight the need for focused special research efforts.

Specific recommendations from the NHLBI Working Group on the Microbiome in Cardiovascular, Pulmonary and Hematologic Health and Diseases included the need to study (1) the role of the virome in shaping and regulating immunity in health and states of inflammation in HLB disorders, as well as the effect of immune strength and function on the virome; (2) the role of the virome in HLB health and diseases through its effects on endothelium, coagulation, and thrombus formation, particularly its effects on platelets, as well as effects on blood cell growth, differentiation, and function and in blood diseases including hereditary hematologic disorders (e.g., sickle cell disease, thalassemia), myeloproliferative disorders, and myelodysplastic syndromes; and (3) the role of the virome in HLB health and diseases in the context of medical and pharmacological interventions such as transfusion, mechanical intubation, immunosuppression, anti-retroviral therapy, and hematopoietic stem cell transplantation (HSCT).

In the past, NHLBI has supported research on viruses affecting blood transfusion safety and heart or lung health [31–33]. Findings from such research and other studies [34–37] informed policy decisions relating to strategies that prevent transmission of viruses such as HIV and hepatitis B or C through blood as well as cells, tissues, and organs. However, there are many other viral elements present in the blood which are not tested for, of which the most recently identified Giant Blood Marseillevirus and of anelloviruses now detected in almost all individuals are just two examples [38–40]. Transfusion of blood to fetuses and infants whose immune system are in development could potentially have a profound impact on their health, either in a positive or negative manner. It is well recognized that exposure to viruses early in life could have very different health impact compared to similar exposure during adulthood. For example, 90 % of infants exposed to hepatitis B virus (HBV) become chronically infected while 95 % of adults recover completely from HBV infection [41]. Although there are as many as 40,000 transfusions performed annually in populations younger than 1 year old in the USA alone [42], the impact of the infused virome on health, for example in shaping and regulating immunity and tolerance, is largely unknown, emphasizing the need for research in this area.

In addition to the outcome of the Working Group described above, the NHLBI Strategic Visioning, an ongoing process dedicated to shape scientific priorities for the Institute and guide future funding strategies over the next decade, identified virome research as an area for further focus. To help address this need, NHLBI developed an initiative to support studies that evaluate how the human virome interacts with its host and the molecular and physiological mechanisms that govern those interactions. The funding opportunity announcement (FOA), RFA-HL-17-002, entitled, “The Role of the Human Virome in Heart, Lung, and Blood Health and Resilience (R61/R33),” was published on October 5, 2016, with the receipt date of June 24, 2016, or for AIDS Applications: August 9, 2016, and will award grants in fiscal year 2017 [43]. It is hoped that this initiative will attract investigators to not only identify additional virome elements but also more importantly to understand how the human virome may affect HLB health and resilience including potential interactions with the immune system and its effect on inflammation. Elucidation of such mechanisms of interaction may help to eventually develop diagnostic and therapeutic intervention strategies targeted to modifying the virome and its effect on host cells. In the context of HIV infection, virome research could help unravel whether associations between the virome, inflammation, and heart, lung, blood, and sleep comorbidities may be influenced by current ART regimens and timing of their initiation upon diagnosis of HIV infection.

## Abbreviations

AIDS, acquired immunodeficiency syndrome; COPD, chronic obstructive pulmonary disease; CVD, cardiovascular disease; FOA, funding opportunity announcement; HBV, hepatitis B virus; HGV, hepatitis G virus; HIV, human immunodeficiency virus; HLB, heart, lung, and blood; HMP, the NIH Human Microbiome Project; HSC, hematopoietic stem cell; HSCT, hematopoietic stem cell transplantation; IBD, inflammatory bowel disease; NHLBI, National Heart, Lung, and Blood Institute; NIH, National Institutes of Health
